# Soluble urokinase plasminogen activator receptor and interleukin‐6 improves prediction of all‐cause mortality and major adverse cardiovascular events in Type 1 diabetes

**DOI:** 10.1111/joim.20108

**Published:** 2025-07-07

**Authors:** Hashmat Sayed Zohori Bahrami, Peter Godsk Jørgensen, Jens Dahlgaard Hove, Ulrik Dixen, Line Jee Hartmann Rasmussen, Jesper Eugen‐Olsen, Peter Rossing, Magnus T. Jensen

**Affiliations:** ^1^ Department of Clinical and Translational Research Steno Diabetes Center Copenhagen Herlev Denmark; ^2^ Department of Cardiology Copenhagen University Hospital, Amager & Hvidovre Hvidovre Denmark; ^3^ Department of Clinical Medicine Faculty of Health and Medical Sciences University of Copenhagen Copenhagen Denmark; ^4^ Department of Cardiology Copenhagen University Hospital Herlev & Gentofte Herlev Denmark; ^5^ Department of Clinical Research Copenhagen University Hospital Amager & Hvidovre Hvidovre Denmark; ^6^ Department of Psychology & Neuroscience Duke University Durham North Carolina USA; ^7^ William Harvey Research Institute NIHR Barts Biomedical Centre Queen Mary University of London Charterhouse Square London UK

**Keywords:** biomarkers, cardiovascular diseases, diabetes, inflammation

## Abstract

**Background:**

Type 1 diabetes (T1D) increases premature mortality risk, with cardiovascular disease being the leading cause. Chronic inflammation may play a role. Associations between inflammatory biomarkers and mortality are not well‐known in T1D.

**Methods:**

We evaluated a prospective clinical cohort with T1D without known cardiovascular disease. The inflammatory biomarkers soluble‐urokinase‐plasminogen‐activator‐receptor (suPAR) and interleukin‐6 (IL‐6) were measured. Patients were stratified by elevated/low suPAR or IL‐6, or simultaneously elevated suPAR and IL‐6. Primary and secondary endpoints were all‐cause mortality and major adverse cardiovascular events (MACE), respectively. Cox models were adjusted for 10 Steno T1 Risk Engine variables and inflammatory biomarkers. Net reclassification improvement (NRI) and C‐statistics were calculated.

**Results:**

Among 962 participants (52% male, median age 50, median follow‐up 13.1 years), mortality was higher in patients with elevated inflammation: 31% for elevated versus 9% for low suPAR; 30% for elevated versus 11% for low IL‐6; and 50% for simultaneously elevated suPAR and IL‐6 versus 5% for low suPAR and IL‐6. In fully adjusted models, elevated inflammation was associated with mortality (hazard ratios [95% confidence intervals]: suPAR 2.0 [1.4–3.0, *p* < 0.001], IL‐6 1.8 [1.3–2.6; *p *= 0.001], and combined 4.0 [2.3–7.2, *p* < 0.001]) and MACE (suPAR 1.9 [1.4–2.6, *p* < 0.001], IL‐6 1.4 [1.0–1.8, *p *= 0.034], and combined 2.6 [1.7–4.1, *p* < 0.001]). Adding suPAR, IL‐6, and their combination to the Steno T1 Risk Engine improved NRI for mortality by 61%, 53%, and 84%, respectively, whereas C‐statistics improved from 0.808 to 0.829, 0.826, and 0.881, respectively.

**Conclusions:**

suPAR, IL‐6, and especially their combination independently predicts all‐cause mortality and MACE in T1D without known cardiovascular disease.

## Graphical abstract

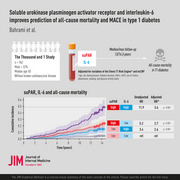

**Abbreviations**
AUC: area under the receiver operating characteristic curve
CI: confidence interval
hsCRP: high‐sensitivity C‐reactive protein
IQRs: interquartile ranges
IL‐6: interleukin‐6
MACE: major adverse cardiovascular events
NRI: net reclassification index
suPAR: soluble urokinase plasminogen activator receptor

## Introduction

Type 1 diabetes (T1D) considerably increases the risk of premature death [1]. Despite advancements in diabetes care, individuals with T1D have roughly 13 years shorter life expectancy compared to the general population [2]. Cardiovascular disease is a leading cause of this excess risk of death [1, 3]. Therefore, it is important to identify individuals with T1D at high risk for all‐cause mortality or cardiovascular disease [4]. Current risk prediction models include various parameters to capture the multifactorial nature of T1D but lack information on inflammation [5]. Recent landmark trials have emphasized the potential of targeting inflammation in T1D [6, 7]. Furthermore, chronic inflammation is a component in the development of atherosclerosis and possibly also heart failure, the main culprits of cardiovascular disease associated with diabetes [8]. Therefore, it holds potential to investigate the prognostic value of biomarkers of chronic inflammation in T1D.

Soluble urokinase plasminogen activator receptor (suPAR) is a biomarker of systemic chronic inflammation [9]. It has been associated with cardiovascular disease in various contexts, highlighting its potential as a predictor of adverse outcomes [10]. Interleukin‐6 (IL‐6), another inflammatory biomarker, has demonstrated therapeutic potential, with its inhibition shown to reduce cardiovascular events, underscoring its role in cardiovascular risk modulation [8, 11, 12]. Despite these findings, the prognostic utility of suPAR and IL‐6 in T1D remains underexplored. This knowledge gap is particularly significant in individuals with T1D without known cardiovascular disease, as this subgroup may benefit from earlier identification of latent cardiovascular risk. Additionally, the incremental value of adding suPAR or IL‐6 to contemporary risk prediction models and their combined utility for risk stratification in T1D remains unexplored.

We aimed to investigate the prognostic value of suPAR and IL‐6 in a cohort of individuals with T1D without known cardiovascular disease. We hypothesized that (A) higher suPAR or higher IL‐6 are associated with an increased risk of all‐cause mortality and adverse cardiovascular events, independent of conventional risk factors, (B) the risk is augmented with the simultaneous increase in both biomarkers, and (C) adding suPAR, IL‐6, or their combination improves the discrimination of an established risk prediction model.

## Methods

### Study population

The Thousand & 1 study is a prospective clinical cohort study of 1093 individuals with T1D without known heart disease [13, 14]. Individuals were included from the Steno Diabetes Center Copenhagen and examined between April 2010 and April 2012. Individuals were eligible if they were 18 years or older, attending the outpatient clinic, willing to participate, and without known heart disease. We defined heart disease as a self‐reported medical history of atrial fibrillation or flutter, heart failure, coronary artery disease (including myocardial infarction, stable angina, percutaneous coronary intervention, or coronary artery bypass surgery), congenital heart disease, pacemaker implantation, or implantable cardioverter defibrillator implantation. Additionally, moderate/severe valvular heart disease and left bundle branch block were clinically assessed at recruitment using echocardiography and electrocardiogram, respectively, following relevant European Society of Cardiology guidelines in effect at the time of inclusion. All of these conditions served as exclusion criteria. Details of the study have been described previously [13, 14]. For the present study, 52 individuals were excluded because of previous stroke diagnosis, and 79 were excluded because of missing suPAR and/or IL‐6 results.

### Biochemistry

All patients initially completed a questionnaire providing information on smoking, exercise, medication use, and diabetes debut. Biochemical data, including HbA1c, creatinine, and albuminuria status, were collected from electronic patient records from the outpatient visit closest to study inclusion, occurring within a maximum of ±4 months from inclusion [13, 14]. HbA1c was measured by high‐performance liquid chromatography (Bio‐Rad Laboratories, Munich, Germany) [13, 14]. Serum creatinine concentration was measured by an enzymatic assay (Hitachi 912; Roche Diagnostics), and the estimated glomerular filtration rate was calculated by the modification of diet in renal disease method [13, 14]. The urinary albumin excretion rate was measured by enzyme immunoassay in 24‐h sterile urine collections [13, 14]. Patients were categorized as normoalbuminuric if the urinary albumin excretion rate, in two out of three consecutive measurements, was <30 mg/24 h, microalbuminuric (moderately increased albuminuria) if the urinary albumin excretion rate was between 30 and 300 mg/24 h, and macroalbuminuria (severely increased albuminuria) if the urinary albumin excretion rate was >300 mg/24 h.

### Inflammatory biomarkers

Serum samples used for suPAR, IL‐6, and high‐sensitive C‐reactive protein (hsCRP) measurements were collected at baseline between 2010 and 2012 and kept frozen at −80°C until measurement in 2022. Samples underwent up to two freeze‐thaw cycles. All three biomarkers are highly stable in frozen samples for long‐term storage.

*Serum suPAR* (ng/mL) was analyzed with the suPARnostic AUTO Flex ELISA (ViroGates A/S) according to the manufacturer's instructions. The assay's detection limit was 0.4 ng/mL, and the upper limit of quantification was 15 ng/mL. The coefficients of variation reported by the manufacturer were 2.8% intra‐assay and 9.2% inter‐assay.

*Serum IL‐6* (pg/mL) was measured with the Human IL‐6 Quantikine ELISA Kit (R&D Systems) according to the manufacturer's instructions. The assay's detection limit was 1.0 pg/mL, and the upper limit of quantification was 300 pg/mL. The coefficients of variation reported by the manufacturer were 1.6%–4.2% intra‐assay and 3.3%–6.4% inter‐assay.

*Serum hsCRP* (mg/L) was measured on a Cobas 6000 analyzer, c501 modules (Roche Diagnostics, GmbH, D‐68298) according to the manufacturer's instructions. The assay's detection limit was 0.15 mg/L. The coefficients of variation reported by the manufacturer were 0.4%–1.6% intra‐assay and 1.3%–8.4% inter‐assay.

### Endpoints and follow‐up

Follow‐up was 100% complete and performed on October 17, 2024, with the registration of data on vital status and hospitalizations from the Danish National Registries. Our primary outcome was all‐cause mortality during follow‐up. As a secondary outcome, we defined a major adverse cardiovascular events (MACE) composite outcome as incident events of hospital admission for ischemic heart disease (ICD‐10: DI200; DI21; DI23‐24), heart failure (ICD‐10: DI110; DI130; DI132; DI42; DI50), stroke (ICD‐10: DI60‐66; DI693‐694; DG45), or all‐cause mortality. Investigators were blinded to endpoints during the acquisition of the inflammatory biomarkers, as the biomarkers were analyzed before collecting outcome data.

### Statistical analysis

Continuous data are presented as medians with interquartile ranges (IQRs) and compared using the Wilcoxon rank‐sum test. Categorical data are presented as numbers (percentages) and compared using the chi‐squared test.

We dichotomized the inflammatory biomarkers (elevated or low) to analyze the associations between two simultaneously elevated biomarkers and outcomes. Because thresholds from large clinical trials are not available for IL‐6 and suPAR, as they are for hsCRP [8], we performed receiver operating characteristics analyses using 12‐year mortality (yes/no) as the outcome and continuous biomarker values as predictors to determine optimal cut‐offs. Cut‐offs for suPAR (≥3.2 ng/mL) and IL‐6 (≥1.9 pg/mL) were identified by maximizing Youden's index [15], and both biomarkers were subsequently dichotomized on the basis of these thresholds. A combination category with elevated chronic inflammation was defined as suPAR ≥ 3.2 ng/mL and IL‐6 ≥ 1.9 pg/mL, and low chronic inflammation was defined as suPAR < 3.2 ng/mL and IL‐6 < 1.9 pg/mL.

We used Cox proportional hazards models to assess the association between suPAR, IL‐6, and their combination with all‐cause mortality or MACE as outcomes. We constructed the following four models: Model 1: crude; Model 2: adjusted for clinically meaningful variables (age, sex, systolic blood pressure, duration of diabetes, HbA1c, estimated glomerular filtration rate, use of statins, and albuminuria status); Model 3: additionally adjusted for lifestyle parameters (smoking [current or prior] and physical activity levels); Model 4: additionally adjusted for the other inflammatory biomarkers. Model 3 was, therefore, equivalent to the Steno T1 Risk Engine [16]. We calculated the variance inflation factor and found no problematic levels of collinearity (variance inflation factor >2) among the model's predictors.

To assess the incremental prognostic value of the biomarkers, we calculated the C‐statistics for the Steno T1 Risk Engine with and without the addition of suPAR, IL‐6, or their combination. Additionally, we calculated the continuous net reclassification index (NRI).

In sensitivity analyses, we further adjusted for left ventricular ejection fraction and hypertension [13]. Hypertension was defined as the usage of one of the following antihypertensive drugs: angiotensin‐converting enzyme inhibitor, angiotensin receptor blocker, beta‐blocker, or calcium antagonist. We dichotomized individuals using two approaches: the upper quartile limits (suPAR ≥ 3.5 ng/mL and IL‐6 ≥ 2.1 pg/mL) and commonly used thresholds distinguishing healthy from non‐healthy individuals (suPAR ≥ 4.0 ng/mL [17] and IL‐6 ≥ 5.2 pg/mL [18]). Additionally, we analyzed the biomarkers as continuous variables to capture their full spectrum of variability. Potential interactions between IL‐6 or suPAR and sex or age (as a continuous variable and stratified at 40, 50, and 60 years) were tested in Cox models. All analyses were also conducted for hsCRP to provide comparative insights.

All suPAR and hsCRP values were above the detection limit, whereas 67% of IL‐6 values were below and imputed to its detection limit (1.0 pg/mL). All model assumptions were validated, including the proportionality of hazards. We present two‐tailed 95% confidence intervals (CI) and *p* values, with *p* < 0.05 considered statistically significant. We used R software, version 4.1.0 (R Project for Statistical Computing, University of Economics and Business Administration, Vienna, Austria).

## Results

### Participants characteristics

This study's final population comprised 962 individuals (52% male; median [IQR] age 50 [40; 60] years). During a maximum follow‐up of 14.5 years (IQR 12.7–14.0), 141 (15%) patients died, and 213 (22%) experienced MACE. Event rates for mortality and MACE per 100 patient years, stratified by biomarker cut‐offs, are presented in Table S1.

suPAR was elevated (≥3.2 ng/mL) in 341 (35%) individuals and IL‐6 (≥1.9 pg/mL) in 261 (27%) individuals. Sensitivity and specificity values for the thresholds in relation to all‐cause mortality are presented in Table S2. Median suPAR was 2.8 ng/mL (IQR 2.3–3.5), and median IL‐6 was 1.0 pg/mL (IQR 1.0–2.1) (Table 1). Individuals with elevated suPAR or elevated IL‐6, in comparison to those with low suPAR or low IL‐6, respectively, were older, had a longer duration of diabetes, higher systolic blood pressure, worse kidney function, and used more medications (Table 1). Individuals with simultaneously elevated suPAR and IL‐6 (*n* = 109) were the oldest, with the highest body mass index, the longest duration of diabetes, worst kidney function, highest HbA1c, frequent smokers, and used most medication (Table S3).

**Table 1:** Clinical characteristics of the study population stratified according to levels of soluble urokinase plasminogen activator receptor (suPAR) and interleukin‐6.

	All	suPAR			Interleukin‐6		
		<3.2 ng/mL	≥3.2 ng/mL	*p* value	<1.9 pg/mL	≥1.9 pg/mL	*p* value
	(*n* = 962)	(*n* = 621)	(*n* = 341)		(*n* = 701)	(*n* = 261)	
Age, years	50 (40; 60)	47 (36; 57)	56 (46; 65)	<0.001	49 (39; 59)	53 (43; 64)	<0.001
Male sex	496 (52)	347 (56)	149 (44)	0.002	361 (51)	135 (52)	0.998
Body mass index, kg/m^2^	25 (23; 28)	25 (23; 28)	25 (23; 28)	0.757	25 (23; 27)	26 (23; 29)	0.008
Diabetes duration, years	26 (15; 36)	22 (13; 32)	33 (19; 44)	<0.001	24 (14; 35)	29 (17; 38)	0.023
Systolic blood pressure, mmHg	130 (120; 140)	130 (120; 140)	140 (120; 150)	<0.001	130 (120; 140)	130 (120; 150)	0.017
eGFR, mL/min/1.73 m^2^	88 (76; 100)	91 (80; 100)	79 (62; 96)	<0.001	89 (77; 100)	86 (71; 100)	0.036
Hemoglobin A1c, mmol/L	65 (57; 74)	64 (56; 73)	66 (58; 76)	0.005	64 (56;73)	66 (58;74)	0.201
Hemoglobin A1c, %	8.1 (7.4; 8.9)	8.0 (7.3; 8.3)	66 (8.2; 9.1)	0.005	8.0 (7.3;8.8)	8.2 (7.5;8.9)	0.201
Total cholesterol, mmol/L	4.8 (4.2; 5.3)	4.8 (4.2; 5.2)	4.8 (4.2; 5.3)	0.647	4.7 (4.2; 5.3)	4.8 (4.3; 5.3)	0.220
Low‐density lipoprotein, mmol/L	2.5 (2.1; 3.0)	2.6 (2.1; 3.0)	2.4 (2.0; 3.0)	0.221	2.5 (2.1; 3.0)	2.5 (2.1; 3.1)	0.529
Left ventricular ejection fraction, %	57 (54; 61)	57 (54; 61)	58 (55; 61)	0.533	58 (54; 61)	57 (54; 61)	0.112
suPAR, ng/mL	2.8 (2.3; 3.5)	2.5 (2.2; 2.8)	4.0 (3.5; 4.8)	<0.001	2.8 (2.3; 3.4)	3.1 (2.5; 3.9)	<0.001
High‐sensitivity CRP, mg/L	14 (6.1; 32)	13 (5.5; 29)	16 (7.6; 28)	0.003	12 (5.2; 25)	25 (11; 56)	<0.001
Interleukin‐6, pg/mL	1.0 (1.0; 2.1)	1.0 (1.0; 1.9)	1.0 (1.0; 3.2)	0.791	1.0 (1.0; 1.0)	5.0 (3.0; 8.7)	<0.001
Current smoking	536 (56)	313 (50)	223 (65)	<0.001	385 (55)	151 (58)	0.718
Albuminuria				<0.001			<0.001
Normoalbuminuria	684 (71)	502 (81)	182 (53)		530 (76)	154 (59)	
Microalbuminuria	191 (20)	97 (16)	94 (28)		124 (18)	67 (26)	
Macroalbuminuria	87 (9)	22 (4)	65 (19)		47 (7)	40 (15)	
Medications at inclusion							
Statins	404 (42)	217 (35)	187 (55)	<0.001	269 (38)	135 (52)	<0.001
ACE‐I/ARB	432 (45)	228 (37)	204 (60)	<0.001	293 (42)	139 (53)	0.006
Beta‐blockers	40 (4)	9 (1)	31 (9)	<0.001	19 (3)	21 (8)	0.001
Calcium antagonists	174 (18)	74 (12)	100 (29)	<0.001	103 (15)	71 (27)	<0.001
Diuretics	245 (25)	100 (16)	145 (43)	<0.001	151 (22)	94 (36)	<0.001

*Note*: Continuous data are reported as medians with IQRs in parentheses. Categorical data are presented as numbers with percentages in parenthesis. *p* value are derived from comparing high and low suPAR and interleukin‐6.

Abbreviations: ACE‐I, angiotensin‐converting enzyme inhibitor; ARB, angiotensin receptor blocker; CRP, C‐reactive protein; eGFR, estimated glomerular filtration rate; IQR, interquartile range; SD, standard deviation; suPAR, soluble urokinase plasminogen activator receptor.

**Table 2:** Incremental prognostic value of soluble urokinase plasminogen activator receptor (suPAR) and interleukin‐6.

	All‐cause mortality		MACE	
	NRI (95% CI) Total Event Non‐event	AUC	NRI (95% CI) Total Event Non‐event	AUC
Steno T1 Risk Engine	Reference	0.808 (0.776–0.840)	Reference	0.761 (0.729–0.793)
Steno T1 Risk Engine + suPAR	61% (44%–79%)		50% (35%–65%)	
	12% (−5% to 29%)	0.829 (0.799–0.859)	5% (−8% to 19%)	0.777 (0.747–0.807)
	49% (43%–55%)		45% (39%–52%)	
Steno T1 Risk Engine + IL‐6	53% (35%–70%)		30% (16%–45%)	
	0% (−17% to 16%)	0.823 (0.793–0.853)	−22% (−35% to −9%)	0.767 (0.737–0.797)
	53% (48%–59%)		52% (46%–58%)	
Steno T1 Risk Engine + suPAR + IL‐6	84% (59%–108%)		51% (31%–71%)	
	14% (−10% to 37%)	0.881 (0.850–0.912)	−17% (−37% to 0%)	0.807 (0.776–0.838)
	70% (64%–77%)		70% (63%–76%)	

*Note*: The Steno T1 Risk Engine includes age, sex, systolic blood pressure, duration of diabetes, HbA1c, estimated glomerular filtration rate, use of statins, albuminuria status, smoking (current or prior), and physical activity levels.

Abbreviations: AUC, area under the receiver operating characteristic curve; CI, confidence interval; IL‐6, interleukin‐6; MACE, major adverse cardiovascular events; NRI, net reclassification index; suPAR, soluble urokinase plasminogen activator receptor.

### Prognostic value of suPAR and IL‐6

**Fig. 1:**
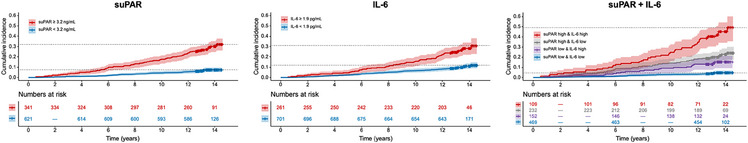
Kaplan–Meier curves for all‐cause mortality stratified by levels of suPAR and IL‐6. The dashed horizontal lines indicate the estimated event probability at the end of the follow‐up. Dashes in risk table (—) indicate that the number at risk is not displayed at that timepoint due to a low number of events between intervals. IL‐6, interleukin‐6; suPAR, soluble urokinase plasminogen activator receptor.

**Fig. 2:**
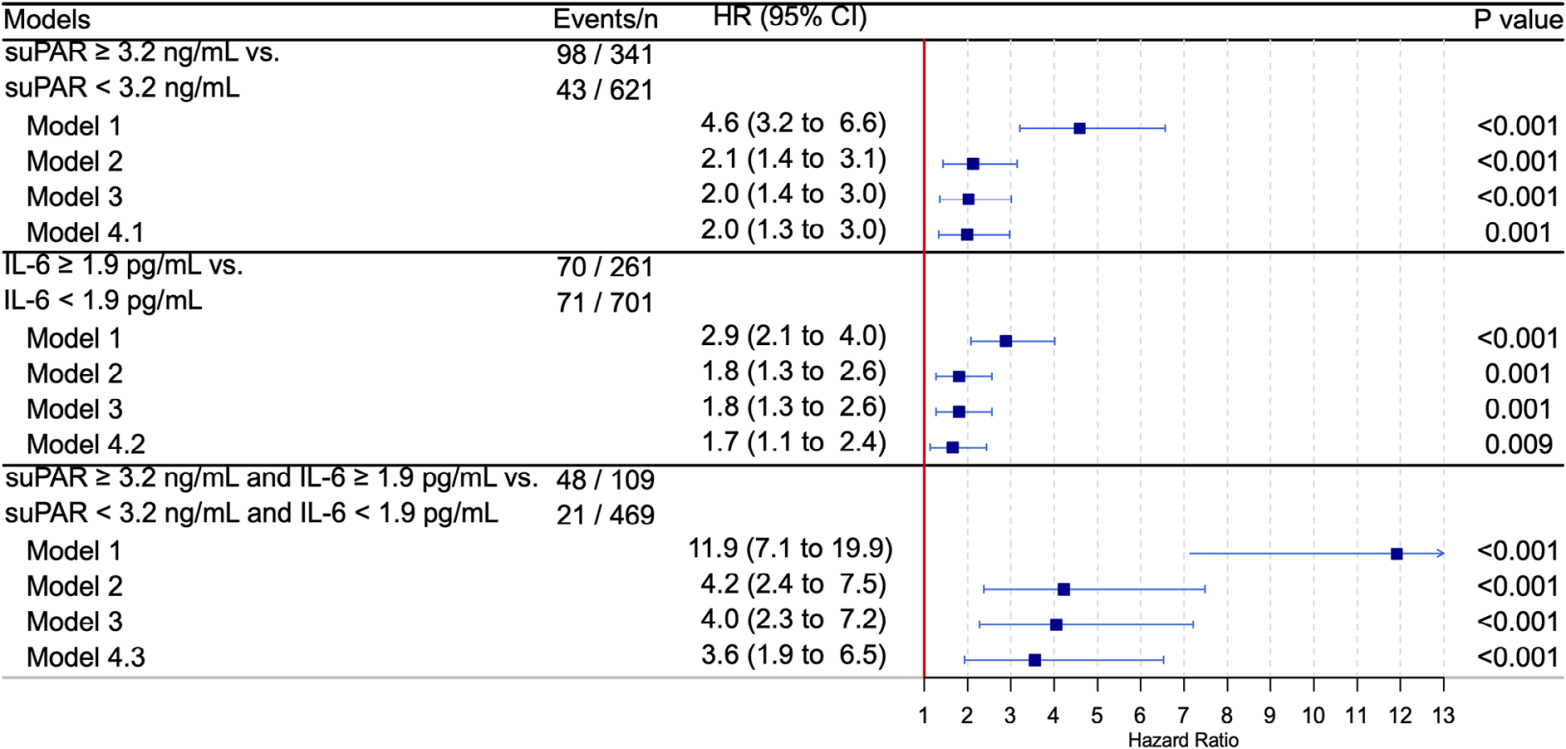
suPAR and IL‐6 and all‐cause mortality in Type 1 diabetes. Model 1: crude, Model 2: adjusted for age, sex, systolic blood pressure, duration of diabetes, HbA1c, estimated glomerular filtration rate, use of statins, and albuminuria status. Model 3: additionally adjusted for smoking (current or prior) and physical activity levels. Model 4.1: additionally adjusted for IL‐6 and hsCRP. Model 4.2: additionally adjusted for suPAR and hsCRP. Model 4.3: additionally adjusted for hsCRP. CI, confidence interval; HR, hazard ratio; hsCRP, high‐sensitivity C‐reactive protein; IL‐6, interleukin‐6; suPAR, soluble urokinase‐type plasminogen activator receptor.

In the univariable analysis, individuals with elevated levels of suPAR or IL‐6 had a ∼30% risk of all‐cause mortality after 14.5 years, compared to ∼10% for those with low suPAR or IL‐6 levels (Fig. 1). Those with a simultaneously elevated suPAR and IL‐6 (*n* = 109, or 11%) had a ∼50% risk of all‐cause mortality after 14.5 years, compared to ∼5% for those with both low suPAR and IL‐6 levels (*n* = 469, or 49%) (Fig. 1). This corresponds to a hazard ratio (HR) of 4.6 for suPAR (95% CI 3.2–6.6, *p* < 0.001), 2.9 for IL‐6 (2.1–4.0, *p* < 0.001), and 11.9 for their combination (7.1–19.9, *p* < 0.001) (Fig. 2). Survival curves for MACE as an outcome and its components are presented in Figs. S1–S4.

**Fig. 3:**
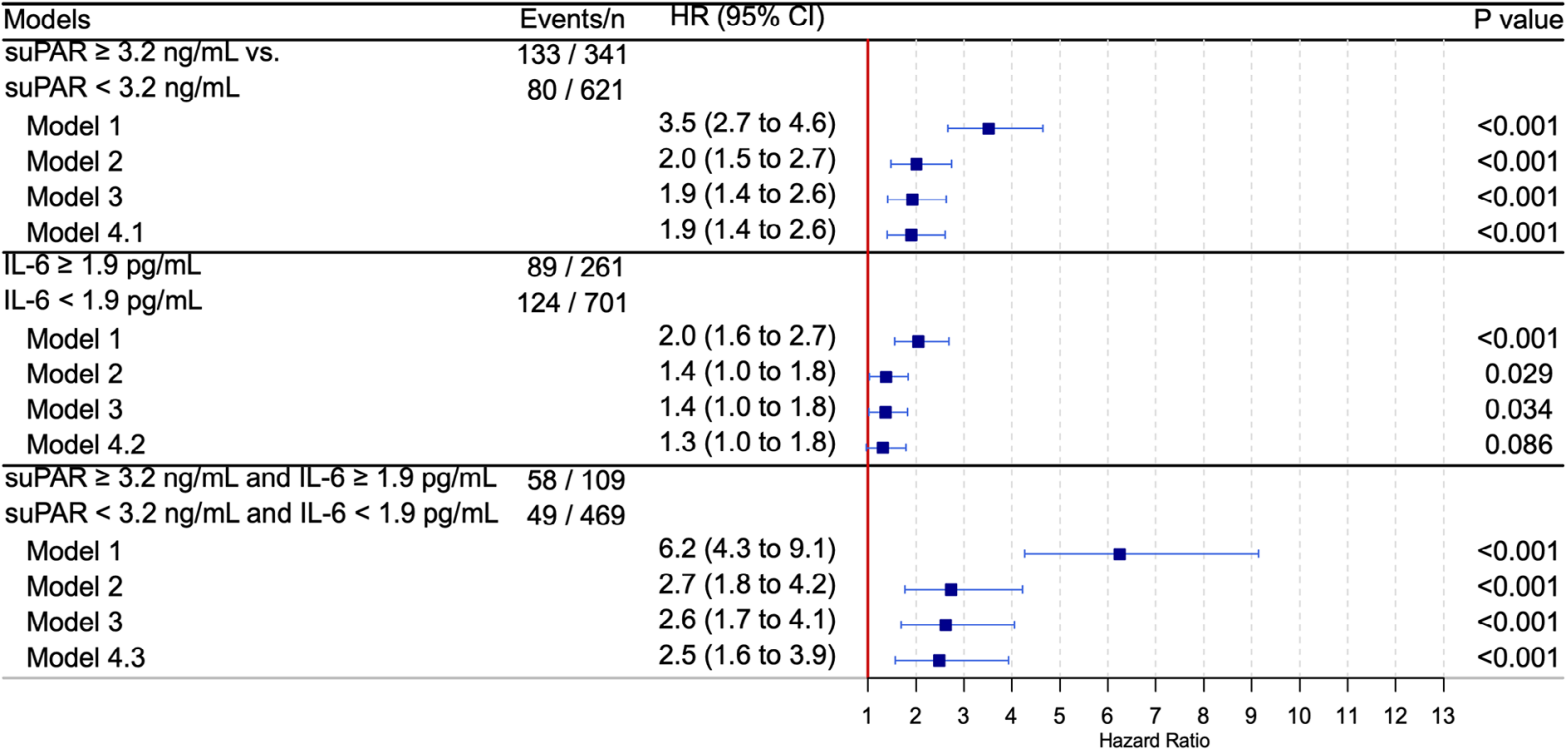
suPAR and IL‐6 and MACE in Type 1 diabetes. Model 1: crude, Model 2: adjusted for age, sex, systolic blood pressure, duration of diabetes, HbA1c, estimated glomerular filtration rate, use of statins, and albuminuria status. Model 3: additionally adjusted for smoking (current or prior) and physical activity levels. Model 4.1: additionally adjusted for IL‐6 and hsCRP. Model 4.2: additionally adjusted for suPAR and hsCRP. Model 4.3: additionally adjusted for hsCRP. CI, confidence interval; HR, hazard ratio; hsCRP, high‐sensitivity C‐reactive protein; IL‐6, interleukin‐6; MACE, major adverse cardiovascular events; suPAR, soluble urokinase‐type plasminogen activator receptor.

In the multivariable analysis, suPAR and IL‐6 remained significantly associated with all‐cause mortality after adjusting for all variables in the Steno T1 Risk Engine (Model 3). The associations persisted when additionally adjusting for the other inflammatory biomarkers, suggesting independent effects of suPAR and IL‐6; suPAR: HR 2.0 (1.3–3.0, *p *= 0.001), IL‐6: HR 1.7 (1.1–2.4, *p *= 0.009) (Fig. 2). The results were similar with MACE as outcome (Model 4), suPAR: HR 1.9 (CI 1.4–2.6, *p* < 0.001), IL‐6: HR 1.3 (1.0–1.8, *p *= 0.086) (Fig. 3). For IL‐6, when adjusting for suPAR but not hsCRP, the HR was 1.4 (1.0–1.8, *p *= 0.037).

Combining suPAR and IL‐6 strengthened the association with all‐cause mortality. Thus, the group with simultaneously elevated levels of suPAR and IL‐6 had an HR of 3.6 (1.9–6.5, *p* < 0.001) compared to the group with both low levels of suPAR and IL‐6 (Fig. 2 and Fig. S5). The association with MACE was also stronger when individuals had elevated levels of both biomarkers, with an HR of 2.5 (1.6–3.9, *p* < 0.001) (Fig. 3 and Fig. S6).

### Incremental value of adding suPAR and IL‐6 to the Steno T1 Risk Engine

For predicting all‐cause mortality, the addition of suPAR or IL‐6 to the Steno T1 Risk Engine improved total NRI by 61% (suPAR), 53% (IL‐6), and 84% (suPAR and IL‐6) (Table 2). Area under the receiver operating characteristic curve (AUC) went from 0.808 for the Steno T1 Risk Engine alone to 0.829 (suPAR), 0.826 (IL‐6), and 0.881 (suPAR and IL‐6) (Table 2).

For predicting MACE, the addition of suPAR or IL‐6 to the Steno T1 Risk Engine improved total NRI by 50% (suPAR), 30% (IL‐6), and 51% (suPAR and IL‐6) (Table 2). AUC went from 0.761 for the Steno T1 Risk Engine alone to 0.777 (suPAR), 0.767 (IL‐6), and 0.807 (suPAR and IL‐6) (Table 2).

### Sensitivity analyses

The results remained consistent when other thresholds were used for IL‐6 and suPAR, as detailed in Tables S4 and S5. Additionally, we investigated hsCRP, which demonstrated a weaker association with all‐cause mortality than suPAR or IL‐6 (Table S6). Tables S4–S6 consistently show the best results when combining suPAR with another biomarker, particularly IL‐6. Furthermore, when analyzing suPAR and IL‐6 as continuous variables, their association remained significant with all‐cause mortality, and their addition to the Steno T1 Risk Engine also improved the NRI and *C*‐statistics (Table S7). Tables S4–S7 also show that the associations were independent of left ventricular ejection fraction and hypertension. No significant interactions were observed between IL‐6 or suPAR and sex or age (tested continuously and stratified at 40, 50, and 60 years).

## Discussion

This prospective study assessed the long‐term prognostic value of suPAR, IL‐6, and their combination regarding all‐cause mortality and MACE in a large (*n* = 962) population of individuals with T1D without known cardiovascular diseases from the Capital Region of Denmark. Our main findings were first, in the unadjusted analysis, individuals with elevated levels of suPAR or IL‐6 had a ∼30% risk of all‐cause mortality compared to ∼10% in individuals with low levels of suPAR or IL‐6. Second, the association with all‐cause mortality (or MACE) was independent of conventional risk factors. Third, each biomarker provided additive information to the other biomarker, such that the risk was substantially higher in individuals with elevated levels of both suPAR and IL‐6; they had, in unadjusted analysis, a ∼50% increased risk of all‐cause mortality compared to ∼5% when both biomarkers were low. Fourth, suPAR, IL‐6, and especially their combination all added incremental value to the Steno T1 Risk Engine.

Our results suggested that measuring markers of chronic inflammation could improve prognostication of all‐cause mortality and cardiovascular risk in individuals with T1D. Although this study does not establish causal relationships, potential mechanistic pathways can be speculated. Recent evidence supports a causal role for suPAR in atherosclerosis, partly through its modulation of monocyte function [19]. This aligns with studies demonstrating suPAR's prognostic value for cardiovascular disease and all‐cause mortality across diverse populations [20, 21, 22]. Similarly, Mendelian randomization studies have causally linked IL‐6 signaling to coronary heart disease [23]. These findings, alongside a broader body of evidence implicating inflammation in cardiovascular disease, have provided the rationale for ongoing Phase III clinical trials targeting IL‐6 signaling in atherosclerosis (ClinicalTrials.gov ID NCT05021835) [24, 25].

Although inflammation's role in cardiovascular disease has been known for decades, it has recently gained renewed importance as a target for intervention [8, 24]. However, data on inflammatory biomarkers’ importance and prognostic performance in T1D remain sparse. In a previous study, we investigated suPAR's ability to predict all‐cause mortality or cardiovascular disease in a smaller cohort with shorter follow‐up [26]. Our current study corroborates and extends these findings by demonstrating these associations in individuals without known cardiovascular diseases over a longer follow‐up period (14.5 vs. 6 years).

Regarding IL‐6, a recent landmark study has shown that inhibiting an IL‐6 cytokine family member improves longevity by 25% [27]. IL‐6 has also been causally linked to atherosclerosis [8, 23]. In T1D, IL‐6 has been associated with cardiovascular disease in a cross‐sectional study [28], and in a smaller prospective study (*n* ≈ 200), we previously observed a potential association between IL‐6 and all‐cause and cardiovascular mortality [29]. However, these earlier studies may be limited by modest sample sizes, shorter follow‐up, and potential confounding by including individuals with existing cardiovascular disease. Our current findings address these limitations, providing evidence of long‐term associations in a large cohort without known cardiovascular disease. The consistency across current and previous studies further supports the prognostic relevance of suPAR or IL‐6 in T1D.

The combination of cardiovascular biomarkers representing different pathophysiological pathways has recently demonstrated a greater incremental predictive value than individual biomarkers, particularly with long‐term follow‐up [30, 31]. Likewise, suPAR and IL‐6 represent different immune activation pathways, and their combination warrants investigation. We have previously shown that combining suPAR with IL‐6 shows a stronger association with left ventricular dysfunction than each biomarker alone in T1D [32]. We are unaware of previous studies investigating the prognostic value of elevated suPAR and IL‐6 combined in T1D. This particular combination was strongly associated with all‐cause mortality in the fully adjusted model (HR 3.6) compared to elevated suPAR (HR 2.0) or IL‐6 (HR 1.7) alone. Additionally, the results of one biomarker were independent of the other biomarker. Thus, these two biomarkers may indicate distinct pathophysiological pathways that interact with each other. The HR 3.6 indicates a profound link between elevated inflammatory biomarkers and heightened mortality risk. Additionally, in our population, the combined use of suPAR and IL‐6 enhanced the discrimination of the Steno T1 Risk Engine, surpassing the improvements observed with each biomarker alone.

The findings suggest that suPAR and IL‐6 may have potential as risk markers in our population of T1D. Thus, two simple blood biomarkers could potentially differentiate between a ∼50% and ∼5% risk of all‐cause mortality when comparing extremes. The 14.5‐year sustained improvement in risk prediction highlights the value of these biomarkers for primary prevention strategies targeting long‐term outcomes beyond the traditional 10‐year estimates. These markers could serve as valuable prognostic tools for the early identification of high‐risk individuals, enabling timely and personalized interventions to reduce mortality and cardiovascular events. Furthermore, these high‐risk individuals may be targets for new treatments, which is particularly intriguing in light of contemporary phase three trials of anti‐IL‐6 drugs for the treatment of major complications of diabetes, such as atherosclerotic diseases (ClinicalTrials.gov ID NCT05021835) and heart failure (ClinicalTrials.gov ID NCT05636176), and a Phase II trial of anti‐suPAR treatment for treating kidney diseases (ClinicalTrials.gov ID NCT06466135). The benefits of targeting inflammation are further supported by large trials showing that inhibiting inflammation reduces cardiovascular risk in high‐risk individuals [8, 33, 34].

Although many other inflammatory markers, including hsCRP, are associated with adverse outcomes, IL‐6 and suPAR may be superior for several reasons. Unlike hsCRP, both suPAR and IL‐6 remained associated with the outcomes in this study independent of other inflammatory biomarkers. Additionally, in contrast to hsCRP [35], the causal role of both suPAR [19] and IL‐6 [23] with atherosclerosis has been established. Both biomarkers are also, unlike other inflammatory biomarkers, being investigated as therapeutic targets in ongoing cardiovascular and kidney trials.

Although hsCRP is a well‐established cardiovascular risk marker, it is a nonspecific acute‐phase reactant that can be elevated in a wide range of transient conditions, such as infections, autoimmune disease flare‐ups, or trauma. Thus, a single baseline measurement of hsCRP cannot differentiate between individuals with chronic low‐grade inflammation and those experiencing a transient inflammatory spike, which increases the risk of misclassification. Combining hsCRP with other biomarkers, such as suPAR or IL‐6, that are more stable over time increases the likelihood of capturing chronic inflammation.

This study underscores the untapped potential of these biomarkers as intervention targets for addressing the myocardial impairment associated with diabetes, particularly T1D. This study also contributes to the growing body of literature on the interplay between diabetes, chronic inflammation, and cardiovascular disease, highlighting the need for care approaches in T1D that also address chronic inflammation [4, 6, 7, 8, 19, 33, 34, 36, 37].

### Strength and limitations

The major strengths of this study are the large sample size, equal distribution of males and females, planned long‐term follow‐up, and the fact that each individual was clinically examined, which enhances the reliability of our findings. Additionally, the study's population consists of individuals without known cardiovascular diseases, which reduces confounding variables and allows for a more focused analysis of suPAR and IL‐6. This strengthens the study's internal validity and increases its generalizability to similar populations without known cardiovascular diseases, which constitute the majority of individuals with T1D. Furthermore, the use of well‐established inflammatory markers adds to the validity of our results. However, several limitations must be acknowledged. First, the possibility of residual confounding cannot be ruled out. Second, cross‐validation was not used to mitigate over‐optimism in the NRI and *C*‐statistics analyses results. Third, the study's single‐center design and predominantly Danish population from the capital region limit generalizability to other ethnicities and regions. Fourth, 67% of IL‐6 values were below the assay's detection limit, and we had to do data imputation, which may introduce errors and uncertainties. However, model assumptions remained valid despite the imputation of IL‐6 values. Additionally, sensitivity analyses using different IL‐6 cut‐offs yielded comparable results, and these cut‐offs would have been the same regardless of how the missing values were imputed, supporting the robustness of our findings. Finally, we did not obtain repeated biomarker measures.

## Conclusion

Elevated biomarkers of chronic inflammation independently predict all‐cause mortality and incident cardiovascular events over 14.5 years in individuals with T1D without known cardiovascular disease from the Capital Region of Denmark. The combination of suPAR and IL‐6 showed the strongest associations, surpassing those observed for either biomarker alone. Although both suPAR and IL‐6 were strong individual predictors, suPAR demonstrated the strongest association. These inflammatory biomarkers add incremental prognostic information to an established risk prediction model. Beyond implications for diagnostics and targeted therapy selection, these data may indicate a role for chronic subclinical inflammation in the pathogenesis of adverse events in T1D. Future research should investigate interventions for individuals with T1D and elevated suPAR and IL‐6.

## Author contributions

**Hashmat Sayed Zohori Bahrami**: Conceptualization; investigation; funding acquisition; writing—original draft; methodology; validation; visualization; software; formal analysis; project administration; data curation; writing—review and editing. **Peter Godsk Jørgensen**: Supervision; writing—review and editing; conceptualization; methodology; investigation. **Jens Dahlgaard Hove**: Writing—review and editing; resources; supervision. **Ulrik Dixen**: Writing—review and editing; supervision; resources. **Line Jee Hartmann Rasmussen**: Methodology; writing—review and editing; supervision; resources; investigation. **Jesper Eugen‐Olsen**: Methodology; writing—review and editing; supervision; resources; investigation. **Peter Rossing**: Conceptualization; investigation; funding acquisition; methodology; writing—review and editing; project administration; data curation; supervision; resources. **Magnus T. Jensen**: Conceptualization; investigation; funding acquisition; methodology; writing—review and editing; project administration; data curation; supervision; resources.

## Funding information

The European Foundation for the Study of Diabetes and the Danish Heart Foundation funded the Thousand & 1 Study. Research grants from Novo Nordisk and the Faculty of Health and Medical Sciences of the University of Copenhagen funded Dr. Bahrami.

## Conflict of interest statement

Peter Godsk Jørgensen reports support for attending two meetings from Medtronic. Jens Dahlgaard Hove reports honoraria for lectures from Novo Nordisk and support for attending two meetings from Novo Nordisk and Astra Zeneca. Jesper Eugen‐Olsen is the chief scientific officer and shareholder in ViroGates A/S and is named inventor of patents using suPAR. Copenhagen University Hospital Hvidovre, Denmark, owns the patents. No patents include work from the current manuscript. Peter Rossing reports grants to his institution from Astra Zeneca, Bayer, and Novo Nordisk and has received honoraria and consulting fees to his institution from Abbott, Astellas, Astra Zeneca, Bayer, Boehringer Ingelheim, Eli Lilly, Gilead, Novo Nordisk, and Sanofi. Magnus T. Jensen is the chief executive officer of Steno Diabetes Center Copenhagen, a public hospital funded through a partnership between the Capital Region of Denmark and the Novo Nordisk Foundation. All other authors have declared no conflicts of interest.

## Disclosure

The sponsors had no role in the study design, data collection, analysis, interpretation, or article writing.

## Ethics statement

The study was performed per the 2nd Helsinki Declaration, approved by the regional ethics committee (H‐3‐2009‐139 and H‐21058624) and the Danish Data Protection Agency (00934‐Geh‐2010‐003 and P‐2021‐719).

## Consent

All participants provided written informed consent [13, 14].

## Supporting information




**Supplemental Figure 1**: Kaplan‐Meier curves for major adverse cardiovascular events stratified by levels of suPAR and interleukin‐6.
**Supplemental Figure 2**: Kaplan‐Meier curves for ischemic heart disease hospitalization stratified by levels of suPAR and interleukin‐6.
**Supplemental Figure 3**: Kaplan‐Meier curves for heart failure hospitalization stratified by levels of suPAR and interleukin‐6.
**Supplemental Figure 4**: Kaplan‐Meier curves for stroke hospitalization stratified by levels of suPAR and interleukin‐6.
**Supplemental Figure 5**: suPAR and interleukin‐6 and all‐cause mortality in type 1 diabetes.
**Supplemental Figure 6**: suPAR and interleukin‐6 and MACE in type 1 diabetes.
**Supplemental Table 1**: Mortality and MACE rates stratified by biomarker levels.
**Supplemental Table 2**: Sensitivity and specificity values for different biomarker thresholds for all‐cause mortality.
**Supplemental Table 3**: Clinical characteristics of the study population stratified according to levels of suPAR and interleukin‐6.
**Supplemental Table 4**: Hazard ratios, net reclassification improvements, and C‐statistics for different thresholds for suPAR and all‐cause mortality.
**Supplemental Table 5**: Hazard ratios, net reclassification improvements, and C‐statistics for different thresholds for interleukin‐6 and all‐cause mortality.
**Supplemental Table 6**: Hazard ratios, net reclassification improvements, and C‐statistics for different thresholds for hsCRP and all‐cause mortality.
**Supplemental Table 7**: Hazard ratios, net reclassification improvements, and C‐statistics for log(2) of suPAR, IL‐6 and hsCRP and all‐cause mortality.

## Data Availability

The data underlying this article cannot be shared publicly for the privacy of individuals who participated in the study. The data will be shared at a reasonable request to the corresponding author.
