# Supplementary material for: Soluble urokinase plasminogen activator receptor and interleukin‐6 improves prediction of all‐cause mortality and major adverse cardiovascular events in Type 1 diabetes
Source: J Intern Med. 2025 Jul 7;298(3):188–99. doi: 10.1111/joim.20108 (PMC12374762; doi:10.1111/joim.20108)

# **Supplemental material**

Supplemental Figure legends

**Supplemental Figure 1**

*Title*: Kaplan-Meier curves for major adverse cardiovascular events stratified by levels of suPAR and interleukin-6

*Footnote:* Dashes in risk table (—) indicate that the number at risk is not displayed at that timepoint, due to a low number of events between intervals.

*Abbreviations:* IL-6 = interleukin-6; suPAR = soluble urokinase-type plasminogen activator receptor.

**Supplemental Figure 2**

*Title*: Kaplan-Meier curves for ischemic heart disease hospitalization stratified by levels of suPAR and interleukin-6

*Footnote:* Dashes in risk table (—) indicate that the number at risk is not displayed at that timepoint, due to a low number of events between intervals.

*Abbreviations:* IL-6 = interleukin-6; suPAR = soluble urokinase-type plasminogen activator receptor.

**Supplemental Figure 3**

*Title*: Kaplan-Meier curves for heart failure hospitalization stratified by levels of suPAR and interleukin-6

*Footnote:* Dashes in risk table (—) indicate that the number at risk is not displayed at that timepoint, due to a low number of events between intervals.

*Abbreviations:* IL-6 = interleukin-6; suPAR = soluble urokinase-type plasminogen activator receptor.

**Supplemental Figure 4**

*Title*: Kaplan-Meier curves for stroke hospitalization stratified by levels of suPAR and interleukin-6

*Footnote:* Dashes in risk table (—) indicate that the number at risk is not displayed at that timepoint, due to a low number of events between intervals.

*Abbreviations:* IL-6 = interleukin-6; suPAR = soluble urokinase-type plasminogen activator receptor.

**Supplemental Figure 5**

*Title*: suPAR and interleukin-6 and all-cause mortality in type 1 diabetes

*Footnote:*

HR from fully adjusted Model 4.3, including age, sex, systolic blood pressure, duration of diabetes, HbA1c, estimated glomerular filtration rate, use of statins, and albuminuria status, smoking (current or prior), physical activity levels, and hsCRP.

*Abbreviations:* CI = confidence interval; HR = hazard ratio; hsCRP = high-sensitivity C-reactive protein; IL-6 = interleukin-6; suPAR = soluble urokinase-type plasminogen activator receptor.

**Supplemental Figure 6**

*Title*: suPAR and interleukin-6 and MACE in type 1 diabetes

*Footnote:*

HR from fully adjusted Model 4.3, including age, sex, systolic blood pressure, duration of diabetes, HbA1c, estimated glomerular filtration rate, use of statins, and albuminuria status, smoking (current or prior), physical activity levels, and hsCRP.

*Abbreviations:* CI = confidence interval; HR = hazard ratio; hsCRP = high-sensitivity C-reactive protein; IL-6 = interleukin-6; MACE = major adverse cardiovascular events; suPAR = soluble urokinase-type plasminogen activator receptor

| **Supplemental Table 1**. Mortality and MACE rates stratified by biomarker levels | | | |
| --- | --- | --- | --- |
| **Groups** | **Events** | **Person-Years** | **Rate pr 100 person years** |
| **All-cause mortality** |  |  |  |
| All | 141 | 12,165 | 1.16 |
| suPAR ≥ 3.2 ng/mL | 98 | 4,090 | 2,40 |
| suPAR < 3.2 ng/mL | 43 | 8,075 | 0,53 |
| IL-6 ≥ 1.9 pg/mL | 70 | 3,153 | 2,22 |
| IL-6 < 1.9 pg/mL | 71 | 9,012 | 0,79 |
| **MACE** |  |  |  |
| All | 213 | 11,178 | 1,91 |
| suPAR ≥ 3.2 ng/mL | 133 | 3,522 | 3,78 |
| suPAR < 3.2 ng/mL | 80 | 7,657 | 1,05 |
| IL-6 ≥ 1.9 pg/mL | 89 | 2,867 | 3,10 |
| IL-6 < 1.9 pg/mL | 124 | 8,312 | 1,49 |
| Abbreviations: IL-6; interleukin-6; MACE = major adverse cardiovascular events; suPAR = soluble urokinase plasminogen activator receptor. | | | |

| **Supplemental Table 2**. Sensitivity and specificity values for different biomarker thresholds for all-cause mortality | | | |
| --- | --- | --- | --- |
|  | | Sensitivity | Specificity |
| **suPAR** | |  |  |
| 3.2 ng/mL | 70% | 71% |  |
| 3.5 ng/mL | 56% | 79% |  |
| 4.0 ng/mL | 43% | 87% |  |
| **IL-6** | |  |  |
| 1.9 pg/mL | 50% | 77% |  |
| 2.1 pg/mL | 47% | 78% |  |
| 5.2 pg/mL | 27% | 89% |  |
| **hsCRP** | |  |  |
| 52 mg/L | 24% | 89% |  |
| 32 mg/L | 32% | 77% |  |
| 2 mg/L | 96% | 6% |  |
| Abbreviations: hsCRP = high-sensitivity C-reactive protein; IL-6 = interleukin-6; MACE = major adverse cardiovascular events; suPAR = soluble urokinase plasminogen activator receptor. | | | |

| **Supplemental Table 3.** Clinical characteristics of the study population stratified according to levels of suPAR and interleukin-6 | | | | | | |
| --- | --- | --- | --- | --- | --- | --- |
|  | | **All** | **suPAR <3.2 ng/mL  & IL-6 <1.9 pg/mL** | **suPAR <3.2 ng/mL  & IL-6 ≥1.9 pg/mL** | **suPAR ≥3.2 ng/mL  & IL-6 <1.9 pg/mL -6** | **suPAR ≥3.2 ng/mL  & IL-6 ≥1.9 pg/mL** |
|  | | (n=962) | (n=469) | (n=152) | (n=232) | (n=109) |
| Age, years | | 50 (40; 60) | 46 (36; 56) | 55 (45; 64) | 55 (45; 64) | 58 (49; 67) |
| Male sex | | 496 (52) | 263 (56) | 84 (55) | 98 (42) | 51 (47) |
| Body mass index, kg/m2 | | 25 (23; 28) | 25 (23; 28) | 25 (23; 29) | 24 (22; 27) | 26 (23; 29) |
| Diabetes duration, years | | 26 (15; 36) | 21 (13; 32) | 24 (15; 33) | 32 (17; 42) | 35 (24; 45) |
| Systolic blood pressure, mmHg | | 130 (120; 140) | 130 (120; 140) | 130 (120; 140) | 130 (120; 150) | 140 (130; 150) |
| eGFR, mL/min/1.73m2 | | 88 (76; 100) | 91 (80; 100) | 90 (80; 110) | 80 (66; 96) | 71 (52; 96) |
| Hemoglobin A1c, mmol/L | | 65 (57; 74) | 63 (56; 72) | 64 (55; 74) | 65 (57; 77) | 68 (62; 78) |
| Hemoglobin A1c, % | | 8.1 (7.4; 8.9) | 7.9 (7.3; 8.7) | 8.0 (7.2; 8.9) | 8.1 (7.4; 9.2) | 8.4 (7.8; 9.3) |
| Total cholesterol, mmol/L | | 4.8 (4.2; 5.3) | 4.7 (4.2; 5.2) | 4.9 (4.3; 5.2) | 4.8 (4.3; 5.3) | 4.8 (4.2; 5.4) |
| Low-density lipoprotein, mmol/L | | 2.5 (2.1; 3.0) | 2.6 (2.1; 3.0) | 2.7 (2.1; 3.1) | 2.4 (2.1; 3.0) | 2.4 (2.0; 3.0) |
| Left ventricular ejection fraction, % | | 57 (54; 61) | 58 (54; 61) | 56 (54; 60) | 57 (55; 61) | 58 (54; 62) |
| suPAR, ng/mL | | 2.8 (2.3; 3.5) | 2.5 (2.1; 2.8) | 2.6 (2.3; 2.9) | 3.9 (3.4; 4.7) | 4.2 (3.5; 5.3) |
| Interleukin-6, pg/mL | | 1.0 (1.0; 2.1) | 1.0 (1.0; 1.0) | 4.5 (2.9; 8.3) | 1.0 (1.0; 1.0) | 5.9 (3.5; 8.9) |
| High-sensitivity CRP, mg/L | | 14 (6.1; 32) | 11 (4.8; 24) | 21 (9.6; 49) | 13 (5.7; 27) | 29 (12; 72) |
| Current smoking | | 536 (56) | 237 (51) | 76 (50) | 148 (64) | 75 (69) |
| Albuminuria | | |  |  |  |  |
|  | Normoalbuminuria | 684 (71) | 392 (84) | 110 (72) | 138 (59) | 44 (40) |
|  | Microalbuminuria | 191 (20) | 63 (13) | 34 (22) | 61 (26) | 33 (30) |
|  | Macroalbuminuria | 87 (9) | 14 (3) | 8 (5) | 33 (14) | 32 (29) |
| Medications at inclusion | | |  |  |  |  |
|  | Statins | 404 (42) | 153 (33) | 64 (42) | 116 (50) | 71 (65) |
|  | ACE-I/ARB | 432 (45) | 163 (35) | 65 (43) | 130 (56) | 76 (68) |
|  | Beta-blockers | 40 (4) | 5 (1) | 4 (3) | 14 (6) | 17 (16) |
|  | Calcium antagonists | 174 (18) | 51 (11) | 23 (15) | 52 (22) | 48 (44) |
|  | Diuretics | 245 (25) | 66 (14) | 34 (22) | 85 (37) | 60 (55) |
| *Footnote: Continuous data are reported as medians with IQRs in parenthesis. Categorical data are presented as numbers with percentages in parenthesis.*  *Abbreviations:* ACE-I = angiotensin-converting enzyme inhibitor; ARB = angiotensin receptor blocker; CRP = C-reactive protein; eGFR = estimated glomerular filtration rate; IQR = interquartile range; SD = standard deviation; suPAR = soluble urokinase plasminogen activator receptor. | | | | | | |

| **Supplemental Table 4.** Hazard ratios, net reclassification improvements, and C-statistics for different thresholds for suPAR and all-cause mortality | | | | | | |
| --- | --- | --- | --- | --- | --- | --- |
|  |  | **Univariate HR** | **Multivariable-adjusted HR*** | **Biomarker-adjusted HR^†^** | **NRI^‡^** | **C-statistics^§^** |
| **Thresholds based on Youden's index** | n |  |  |  |  |  |
| suPAR ≥ 3.2 ng/mL | 341 | 4.6 (3.2 to 6.6) | 2.1 (1.4 to 3.2) | 2.1 (1.4 to 3.1) | 61% (44% to 79%) | 0.829 (0.799 to 0.859) |
| suPAR ≥ 3.2 ng/mL and IL-6 ≥ 1.9 pg/mL | 109 | 12.1 (7.2 to 20.2) | 2.8 (1.6 to 4.8) | 2.8 (1.6 to 4.8) | 84% (59% to 108%) | 0.881 (0.850 to 0.912) |
| suPAR ≥ 3.2 ng/mL and hsCRP ≥ 52 mg/L | 59 | 8.1 (4.8 to 13.7) | 3.8 (1.9 to 7.6) | 3.3 (1.5 to 6.9) | 84% (57% to 112%) | 0.881 (0.847 to 0.915) |
| **Thresholds based on upper quartiles** |  |  |  |  |  |  |
| suPAR ≥ 3.5 ng/mL | 252 | 4.0 (2.9 to 5.5) | 1.8 (1.2 to 2.6) | 1.8 (1.2 to 2.6) | 56% (38% to 73%) | 0.826 (0.795 to 0.857) |
| suPAR ≥ 3.5 ng/mL and IL-6 ≥ 2.1 pg/mL | 83 | 9.2 (5.9 to 14.3) | 3.5 (1.9 to 6.5) | 4.4 (2.3 to 8.3) | 74% (51% to 97%) | 0.874 (0.837 to 0.911) |
| suPAR ≥ 3.5 ng/mL and hsCRP ≥ 32 mg/L | 74 | 5.1 (3.2 to 8.3) | 2.1 (1.1 to 3.8) | 2.0 (1.0 to 3.9) | 74% (50% to 98%) | 0.855 (0.819 to 0.891) |
| **Thresholds based on the literature** |  |  |  |  |  |  |
| suPAR ≥ 4.0 ng/mL | 166 | 4.2 (3.0 to 5.8) | 1.7 (1.2 to 2.6) | 1.6 (1.1 to 2.5) | 55% (38% to 73%) | 0.826 (0.795 to 0.857) |
| suPAR ≥ 4.0 ng/mL and IL-6 ≥ 5.2 pg/mL | 42 | 9.1 (5.7 to 14.3) | 2.8 (1.5 to 5.1) | 2.8 (1.5 to 5.1) | 29% (8% to 50%) | 0.842 (0.805 to 0.879) |
| suPAR ≥ 4.0 ng/mL and hsCRP ≥ 2 mg/L | 157 | 6.6 (2.6 to 16.4) | 2.1 (0.8 to 5.9) | 1.9 (0.7 to 5.3) | 4% (-24% to 32%) | 0.772 (0.712 to 0.832) |
| *Footnotes:* References are: row 1 suPAR <3.2, row 2 suPAR <3.2 and IL-6 <1.9, row 3 suPAR <3.2 and hsCRP <52. The same logic applies to rows 4 through 9.  Numbers in parenthesis denotes 95% confidence intervals.  *: Adjusted for clinical variables included in the Steno T1 Risk Engine: age, sex, systolic blood pressure, duration of diabetes, HbA1c, estimated glomerular filtration rate, use of statins, albuminuria status, smoking (current or prior), physical activity levels, left ventricular ejection fraction, and hypertension.  †: Multivariable-adjusted* and additionally adjusted for: 1^st^, 4^th^ and 7^th^ row IL-6 and hsCRP; 2^nd^, 5^th^, and 8^th^ row hsCRP; 3^rd,^ 6^th^, and 9^th^ row IL-6.  ‡: Improvement when adding the biomarker(s) to a baseline model containing all the variables from the Steno T1 Risk Engine*  §: C-statistic of baseline model containing variables from the Steno T1 Risk Engine* = 0.808 with 95% confidence intervals 0.776 to 0.840.  *Abbreviations:* HR = Hazard ratio; hsCRP = high-sensitivity C-reactive protein; L-6 = interleukin-6; NRI = net reclassification improvement; suPAR = soluble urokinase plasminogen activator receptor. | | | | | | |

| **Supplemental Table 5.** Hazard ratios, net reclassification improvements, and C-statistics for different thresholds for interleukin-6 and all-cause mortality | | | | | | |
| --- | --- | --- | --- | --- | --- | --- |
|  |  | **Univariate HR** | **Multivariable-adjusted HR*** | **Biomarker-adjusted HR^†^** | **NRI^‡^** | **C-statistics^§^** |
| **Thresholds based on Youden's index** | n |  |  |  |  |  |
| IL-6 ≥ 1.9 pg/mL | 261 | 2.9 (2.1 to 4.0) | 1.8 (1.3 to 2.5) | 1.7 (1.1 to 2.4) | 53% (35% to 70%) | 0.826 (0.795 to 0.857) |
| IL-6 ≥ 1.9 pg/mL and suPAR ≥ 3.2 ng/mL | 109 | 12.1 (7.2 to 20.2) | 2.8 (1.6 to 4.8) | 2.8 (1.6 to 4.8) | 84% (59% to 108%) | 0.881 (0.850 to 0.912) |
| IL-6 ≥ 1.9 pg/mL and hsCRP ≥ 52 mg/L | 76 | 4.5 (2.9 to 6.9) | 2.4 (1.5 to 4.0) | 2.3 (1.4 to 3.8) | 33% (12% to 53%) | 0.828 (0.792 to 0.864) |
| **Thresholds based on upper quartiles** |  |  |  |  |  |  |
| IL-6 ≥ 2.1 pg/mL | 247 | 2.8 (2 .0to 3.9) | 1.8 (1.2 to 2.5) | 1.7 (1.2 to 2.5) | 47% (30% to 65%) | 0.826 (0.795 to 0.857) |
| IL-6 ≥ 2.1 pg/mL and suPAR ≥ 3.5 ng/mL | 83 | 9.2 (5.9 to 14.3) | 3.5 (1.9 to 6.5) | 4.4 (2.3 to 8.3) | 74% (51% to 97%) | 0.874 (0.837 to 0.911) |
| IL-6 ≥ 2.1 pg/mL and hsCRP ≥ 32 mg/L | 106 | 3.3 (2.2 to 5.0) | 2.0 (1.3 to 3.2) | 2.0 (1.3 to 3.2) | 32% (11% to 53%) | 0.822 (0.786 to 0.858) |
| **Thresholds based on the literature** |  |  |  |  |  |  |
| IL-6 ≥ 5.2 pg/mL | 127 | 2.7 (1.9 to 4.0) | 1.7 (1.1 to 2.6) | 1.6 (1.1 to 2.5) | 48% (30% to 65%) | 0.823 (0.791 to 0.855) |
| IL-6 ≥ 5.2 pg/mL and suPAR ≥ 4.0 ng/mL | 42 | 9.1 (5.7 to 14.3) | 2.8 (1.5 to 5.1) | 2.8 (1.5 to 5.1) | 29% (8% to 50%) | 0.842 (0.805 to 0.879) |
| IL-6 ≥ 5.2 pg/mL and hsCRP ≥ 2 mg/L | 122 | 4.0 (1.8 to 8.9) | 1.6 (0.6 to 4.1) | 1.3 (0.5 to 3.3) | 39% (6% to 72%) | 0.824 (0.760 to 0.888) |
| *Footnote:* References are: row 1 IL-6 <1.9, row 2 IL-6 <1.9 and suPAR <3.2, row 3 IL-6 <1.9 and hsCRP <52. The same logic applies to rows 4 through 9.  Numbers in parenthesis denotes 95% confidence intervals.  *: Adjusted for clinical variables included in the Steno T1 Risk Engine: age, sex, systolic blood pressure, duration of diabetes, HbA1c, estimated glomerular filtration rate, use of statins, albuminuria status, smoking (current or prior), physical activity levels, left ventricular ejection fraction, and hypertension.  †: Multivariable-adjusted and additionally adjusted for: 1^st^, 4^th^ and 7^th^ row suPAR and hsCRP; 2^nd^, 5^th^, and 8^th^ row hsCRP; 3^rd,^ 6^th^, and 9^th^ row suPAR.  ‡: Improvement when adding the biomarker(s) to a baseline model containing all the variables from the Steno T1 Risk Engine*  §: C-statistic of baseline model containing variables from the Steno T1 Risk Engine* = 0.808 with 95% confidence intervals 0.776 to 0.840.  *Abbreviations:* HR = Hazard ratio; hsCRP = high-sensitivity C-reactive protein; L-6 = interleukin-6; NRI = net reclassification improvement; suPAR = soluble urokinase plasminogen activator receptor. | | | | | | |

| **Supplemental Table 6.** Hazard ratios, net reclassification improvements, and C-statistics for different thresholds for hsCRP and all-cause mortality | | | | | | |
| --- | --- | --- | --- | --- | --- | --- |
|  |  | **Univariate HR** | **Multivariable-adjusted HR*** | **Biomarker-adjusted HR^†^** | **NRI^‡^** | **C-statistics^§^** |
| **Thresholds based on Youden's index** | n |  |  |  |  |  |
| hsCRP ≥ 52 mg/L | 123 | 2.3 (1.6 to 3.4) | 1.8 (1.2 to 2.8) | 1.3 (0.8 to 2.1) | 38% (21% to 55%) | 0.827 (0.797 to 0.857) |
| hsCRP ≥ 52 mg/L and suPAR ≥ 3.2 ng/mL | 59 | 8.1 (4.8 to 13.7) | 3.8 (1.9 to 7.6) | 3.3 (1.5 to 6.9) | 84% (57% to 112%) | 0.881 (0.847 to 0.915) |
| hsCRP ≥ 52 mg/L and IL-6 ≥ 1.9 pg/mL | 76 | 4.5 (2.9 to 6.9) | 2.4 (1.5 to 4.0) | 2.3 (1.4 to 3.8) | 33% (12% to 53%) | 0.828 (0.792 to 0.864) |
| **Thresholds based on upper quartiles** |  |  |  |  |  |  |
| hsCRP ≥ 32 mg/L | 235 | 1.5 (1.0 to 2.1) | 1.3 (0.9 to 1.9) | 1.0 (0.7 to 1.6) | 53% (36% to 71%) | 0.821 (0.79 to 0.852) |
| hsCRP ≥ 32 mg/L and suPAR ≥ 3.5 ng/mL | 74 | 5.1 (3.2 to 8.3) | 2.1 (1.1 to 3.8) | 2.0 (1.0 to 3.9) | 74% (50% to 98%) | 0.855 (0.819 to 0.891) |
| hsCRP ≥ 32 mg/L and IL-6 ≥ 2.1 pg/mL | 106 | 3.3 (2.2 to 5.0) | 2.0 (1.3 to 3.2) | 2.0 (1.3 to 3.2) | 32% (11% to 53%) | 0.822 (0.786 to 0.858) |
| **Thresholds based on the literature** |  |  |  |  |  |  |
| hsCRP ≥ 2 mg/L | 869 | 1.5 (0.8 to 2.9) | 0.8 (0.4 to 1.6) | 0.8 (0.4 to 1.5) | 61% (44% to 79%) | 0.821 (0.789 to 0.853) |
| hsCRP ≥ 2 mg/L and suPAR ≥ 4.0 ng/mL | 157 | 6.6 (2.6 to 16.4) | 2.1 (0.8 to 5.9) | 1.9 (0.7 to 5.3) | 4% (-24% to 32%) | 0.772 (0.712 to 0.832) |
| hsCRP ≥ 2 mg/L and IL-6 ≥ 5.2 pg/mL | 122 | 4.0 (1.8 to 8.9) | 1.6 (0.6 to 4.1) | 1.3 (0.5 to 3.3) | 39% (6% to 72%) | 0.824 (0.76 to 0.888) |
| *Footnote:* References are: row 1 hsCRP <52, row 2 hsCRP <52 and suPAR <3.2, row 3 hsCRP <52 and IL-6 <1.9. The same logic applies to rows 4 through 9.  Numbers in parenthesis denotes 95% confidence intervals.  *: Adjusted for clinical variables included in the Steno T1 Risk Engine: age, sex, systolic blood pressure, duration of diabetes, HbA1c, estimated glomerular filtration rate, use of statins, albuminuria status, smoking (current or prior), physical activity levels, left ventricular ejection fraction, and hypertension.  †: Multivariable-adjusted and additionally adjusted for: 1^st^, 4^th^ and 7^th^ row suPAR and IL-6; 2^nd^, 5^th^, and 8^th^ row IL-6; 3^rd,^ 6^th^, and 9^th^ row suPAR.  ‡: Improvement when adding the biomarker(s) to a baseline model containing all the variables from the Steno T1 Risk Engine*  §: C-statistic of baseline model containing variables from the Steno T1 Risk Engine* = 0.808 with 95% confidence intervals 0.776 to 0.840.  *Abbreviations:* HR = Hazard ratio; hsCRP = high-sensitivity C-reactive protein; L-6 = interleukin-6; NRI = net reclassification improvement; suPAR = soluble urokinase plasminogen activator receptor. | | | | | | |

| **Supplemental Table 7.** Hazard ratios, net reclassification improvements, and C-statistics for log(2) of suPAR, IL-6 and hsCRP and all-cause mortality | | | | | |
| --- | --- | --- | --- | --- | --- |
|  | **Univariate HR** | **Multivariable-adjusted HR*** | **Biomarker-adjusted HR^†^** | **NRI^‡^** | **C-statistics^§^** |
| suPAR | 2.4 (2 to 2.8) | 1.9 (1.3 to 2.8) | 1.9 (1.3 to 2.7) | 47% (30% to 65%) | 0.828 (0.797 to 0.859) |
| IL-6 | 1.4 (1.3 to 1.5) | 1.3 (1.1 to 1.4) | 1.2 (1.1 to 1.4) | 46% (29% to 63%) | 0.826 (0.795 to 0.857) |
| hsCRP | 1.2 (1.1 to 1.3) | 1.1 (1.0 to 1.2) | 1.0 (0.9 to 1.1) | 62% (44% to 79%) | 0.820 (0.789 to 0.851) |
| *Footnote:* numbers in parenthesis denotes 95% confidence intervals.  *: Adjusted for clinical variables included in the Steno T1 Risk Engine: age, sex, systolic blood pressure, duration of diabetes, HbA1c, estimated glomerular filtration rate, use of statins, albuminuria status, smoking (current or prior), physical activity levels, left ventricular ejection fraction, and hypertension.  †: Multivariable-adjusted and additionally adjusted for: 1^st^ row IL-6 and hsCRP; 2^nd^ row suPAR and hsCRP; 3^rd^ row IL-6 and suPAR.  ‡: Improvement when adding the biomarker to a baseline model containing all the variables from the Steno T1 Risk Engine*  §: C-statistic of baseline model containing variables from the Steno T1 Risk Engine* = 0.808 with 95% confidence intervals 0.776 to 0.840.  *Abbreviations:* HR = Hazard ratio; hsCRP = high-sensitivity C-reactive protein; L-6 = interleukin-6; NRI = net reclassification improvement; suPAR = soluble urokinase plasminogen activator receptor. | | | | | |


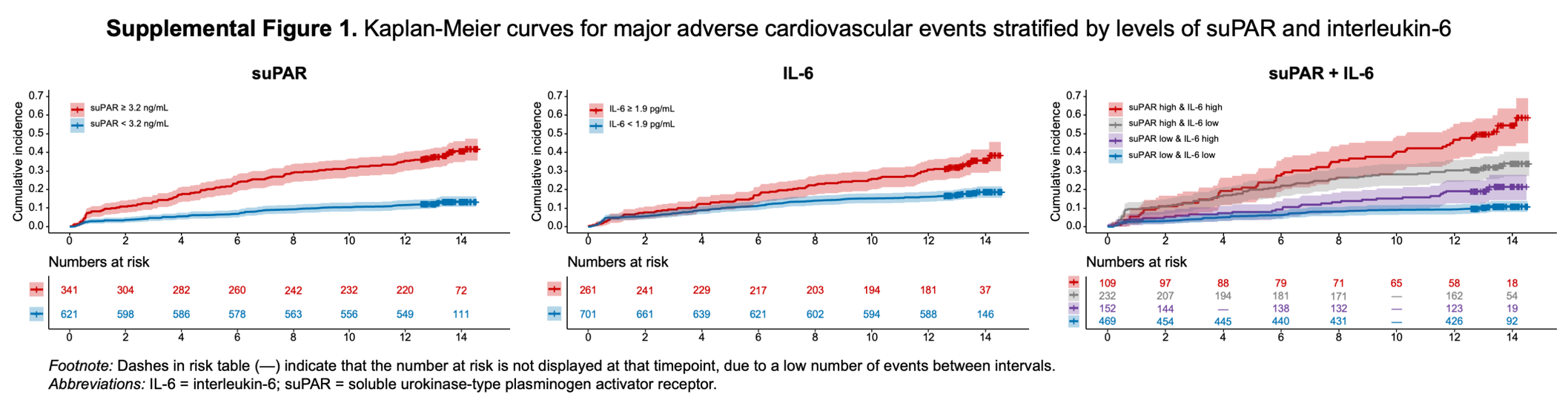


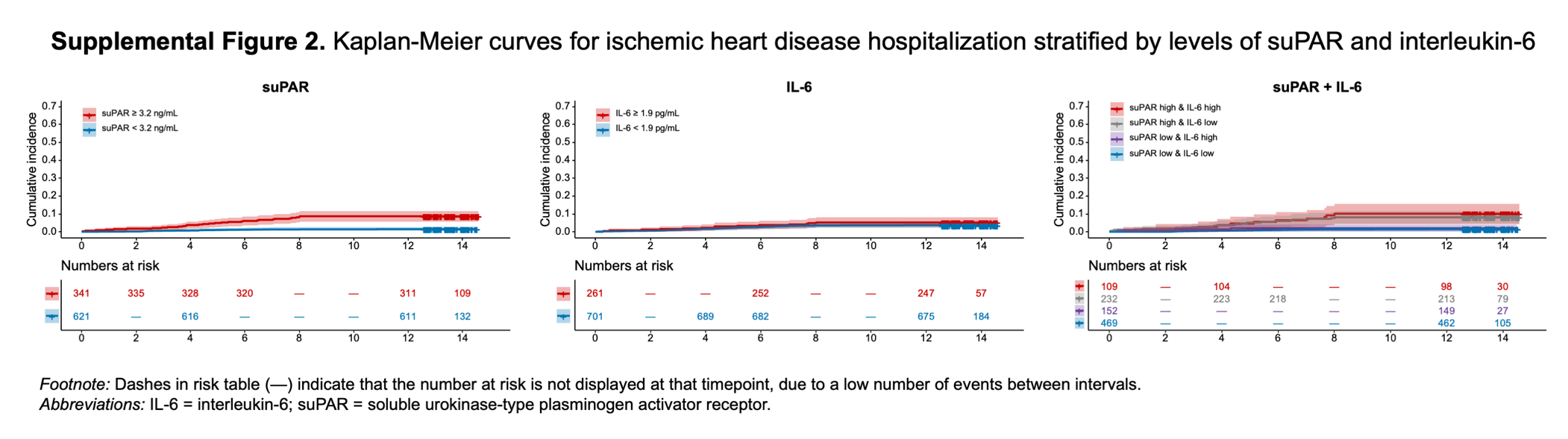


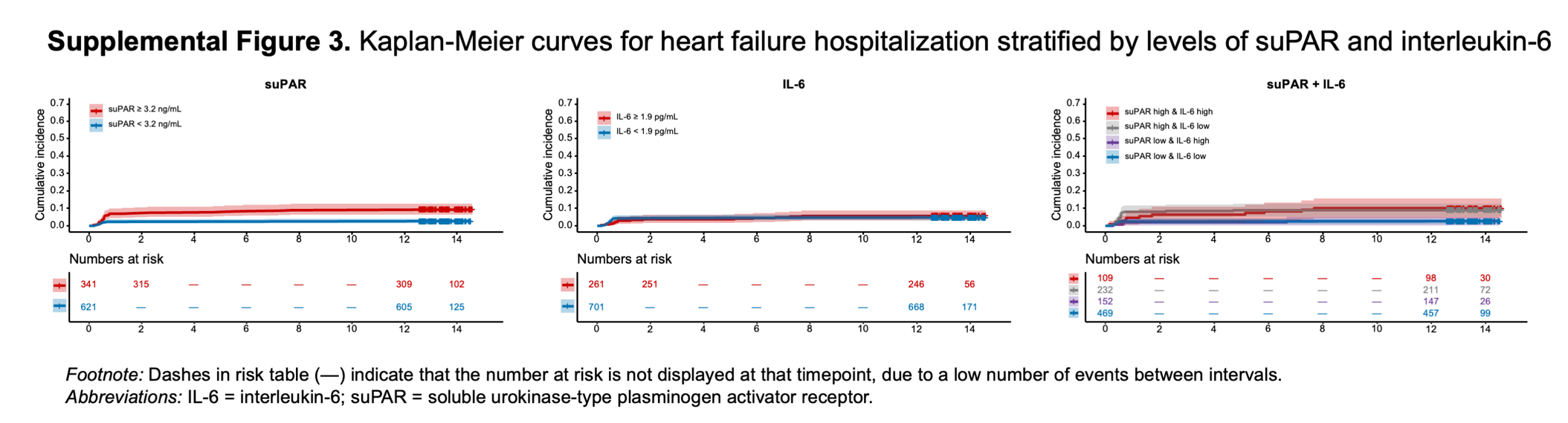


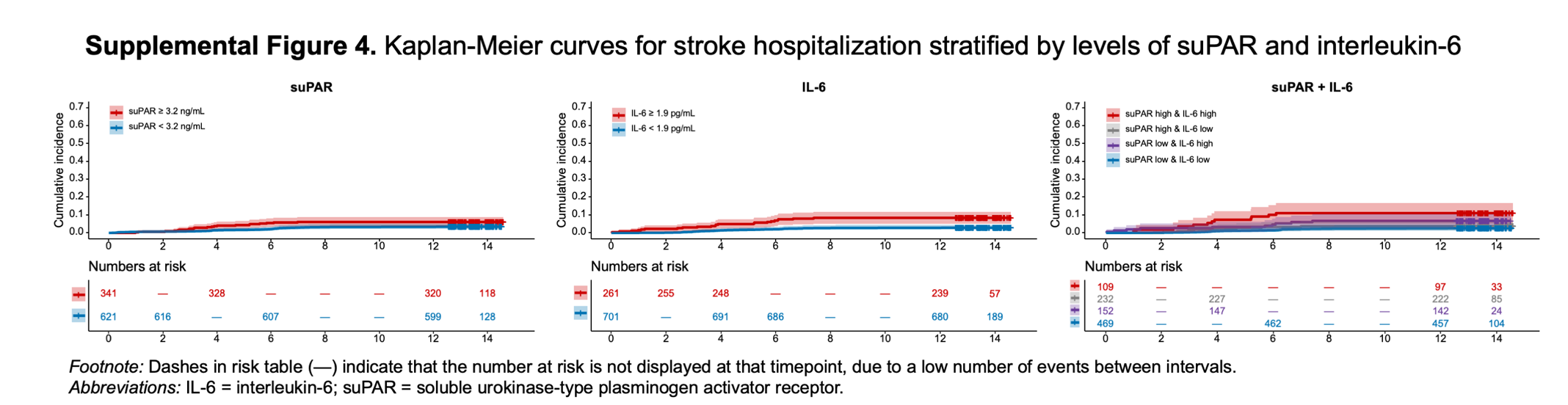


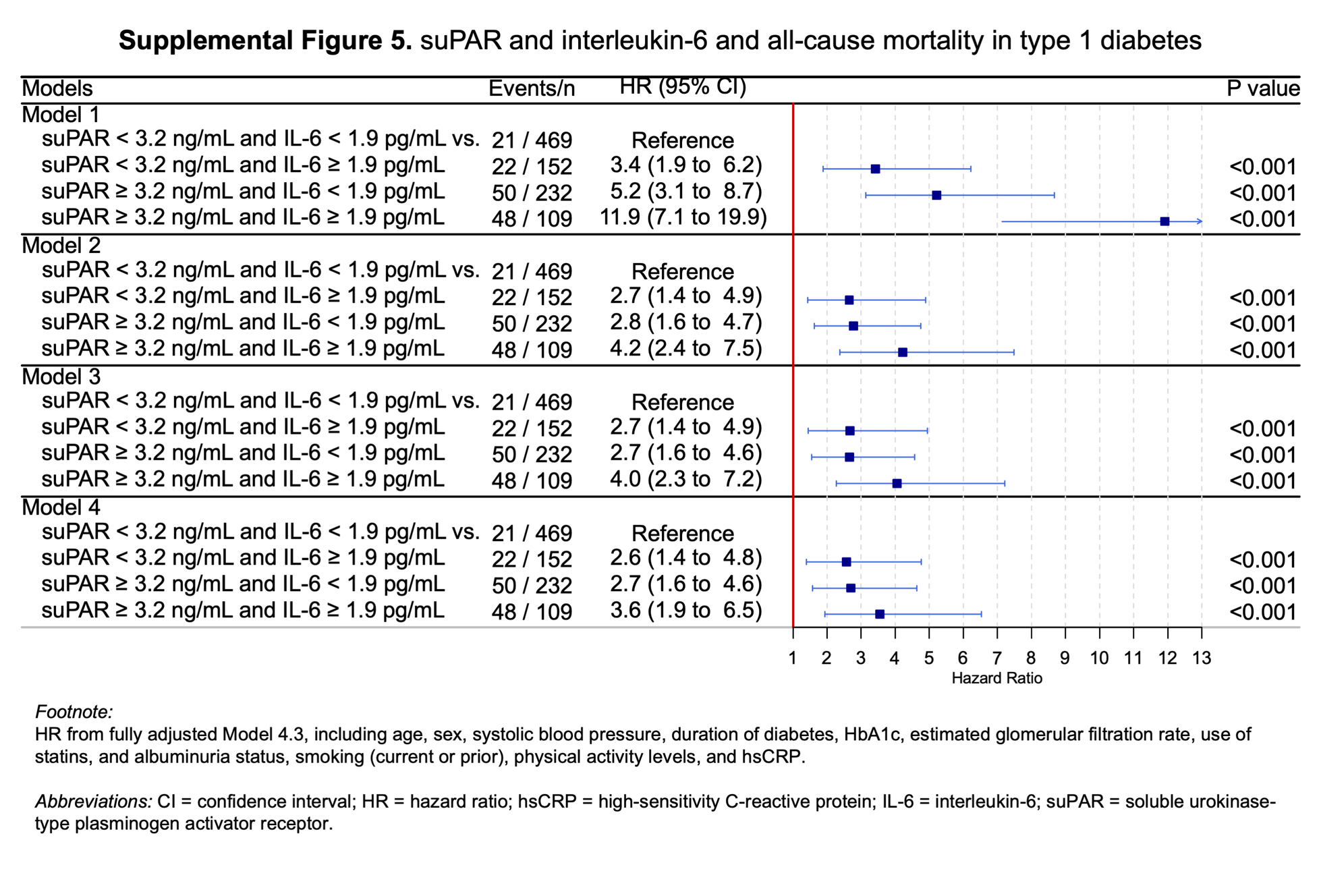


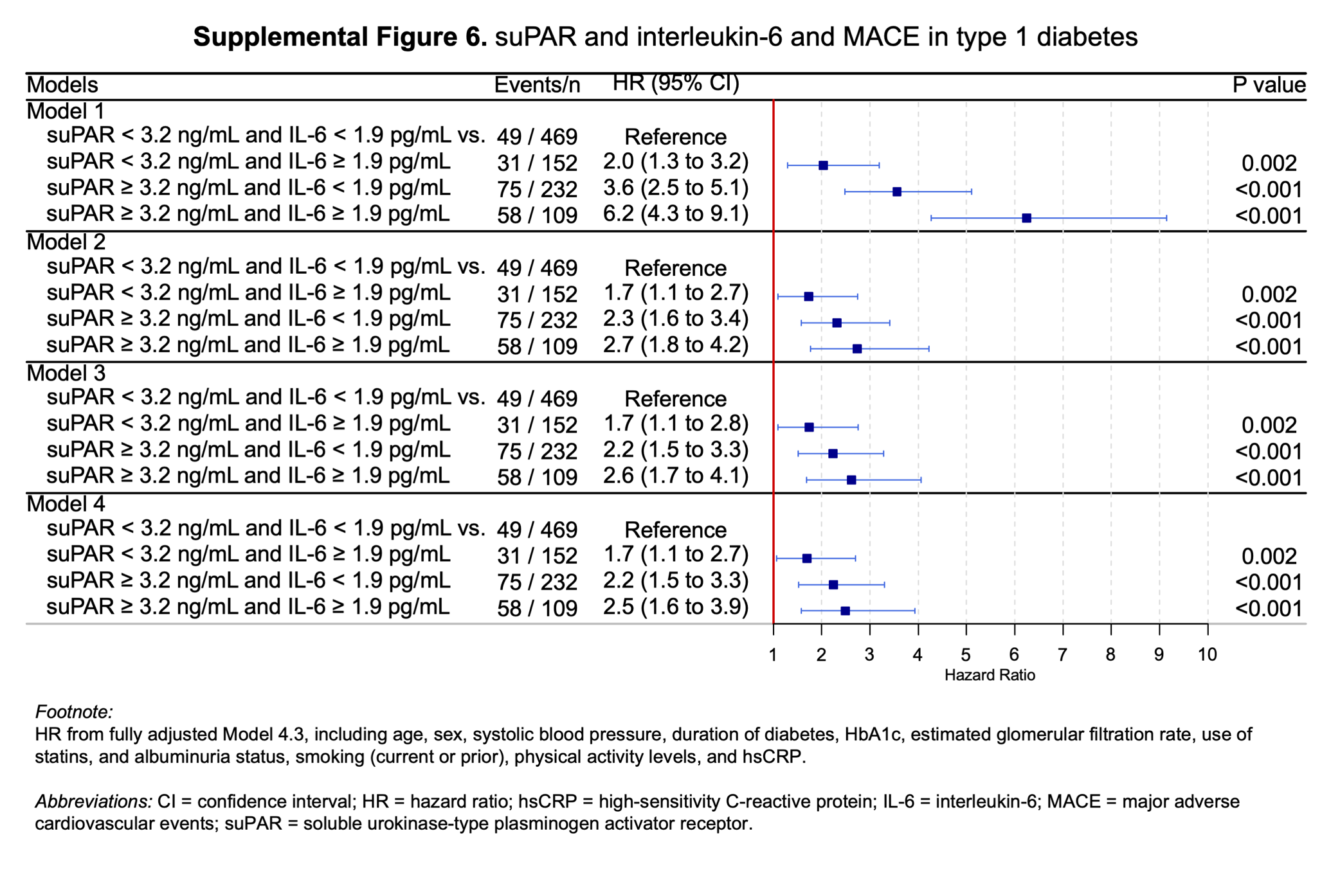

Supplement: Supplementary file 1 — Supplemental Figure 1: Kaplan‐Meier curves for major adverse cardiovascular events stratified by levels of suPAR and interleukin‐6. Supplemental Figure 2: Kaplan‐Meier curves for ischemic heart disease hospitalization stratified by levels of suPAR and interleukin‐6. Supplemental Figure 3: Kaplan‐Meier curves for heart failure hospitalization stratified by levels of suPAR and interleukin‐6. Supplemental Figure 4: Kaplan‐Meier curves for stroke hospitalization stratified by levels of suPAR and interleukin‐6. Supplemental Figure 5: suPAR and interleukin‐6 and all‐cause mortality in type 1 diabetes. Supplemental Figure 6: suPAR and interleukin‐6 and MACE in type 1 diabetes. Supplemental Table 1: Mortality and MACE rates stratified by biomarker levels. Supplemental Table 2: Sensitivity and specificity values for different biomarker thresholds for all‐cause mortality. Supplemental Table 3: Clinical characteristics of the study population stratified according to levels of suPAR and interleukin‐6. Supplemental Table 4: Hazard ratios, net reclassification improvements, and C‐statistics for different thresholds for suPAR and all‐cause mortality. Supplemental Table 5: Hazard ratios, net reclassification improvements, and C‐statistics for different thresholds for interleukin‐6 and all‐cause mortality. Supplemental Table 6: Hazard ratios, net reclassification improvements, and C‐statistics for different thresholds for hsCRP and all‐cause mortality. Supplemental Table 7: Hazard ratios, net reclassification improvements, and C‐statistics for log(2) of suPAR, IL‐6 and hsCRP and all‐cause mortality. [file JOIM-298-188-s001.docx]
